# Unknown Disease Outbreaks Detection: A Pilot Study on Feature-Based Knowledge Representation and Reasoning Model

**DOI:** 10.3389/fpubh.2021.683855

**Published:** 2021-05-13

**Authors:** Rui Feng, Qiping Hu, Yingan Jiang

**Affiliations:** ^1^School of Computer Science, Wuhan University, Wuhan, China; ^2^Department of Infectious Diseases, Renmin Hospital of Wuhan University, Wuhan, China

**Keywords:** knowledge representation, reasoning model, medical diagnosis, medical expert systems, surveillance, unknown disease outbreaks

## Abstract

**Background:** The outbreak of COVID-19 in 2019 has rapidly swept the world, causing irreparable loss to human beings. The pandemic has shown that there is still a delay in the early response to disease outbreaks and needs a method for unknown disease outbreak detection. The study's objective is to establish a new medical knowledge representation and reasoning model, and use the model to explore the feasibility of unknown disease outbreak detection.

**Methods:** The study defined abnormal values with diagnostic significances from clinical data as the Features, and defined the Features as the antecedents of inference rules to match with knowledge bases, achieved in detecting known or emerging infectious disease outbreaks. Meanwhile, the study built a syndromic surveillance base to capture the target cases' Features to improve the reliability and fault-tolerant ability of the system.

**Results:** The study combined the method with Severe Acute Respiratory Syndrome (SARS), Middle East Respiratory Syndrome (MERS), and early COVID-19 outbreaks as empirical studies. The results showed that with suitable surveillance guidelines, the method proposed in this study was capable to detect outbreaks of SARS, MERS, and early COVID-19 pandemics. The quick matching accuracies of confirmed infection cases were 89.1, 26.3–98%, and 82%, and the syndromic surveillance base would capture the Features of the remaining cases to ensure the overall detection accuracies. Based on the early COVID-19 data in Wuhan, this study estimated that the median time of the early COVID-19 cases from illness onset to local authorities' responses could be reduced to 7.0–10.0 days.

**Conclusions:** This study offers a new solution to transfer traditional medical knowledge into structured data and form diagnosis rules, enables the representation of doctors' logistic thinking and the knowledge transmission among different users. The results of empirical studies demonstrate that by constantly inputting medical knowledge into the system, the proposed method will be capable to detect unknown diseases from existing ones and perform an early response to the initial outbreaks.

## Introduction

In December 2019, a series of pneumonia cases with unknown etiologies appeared in Wuhan, Hubei province, China. The clinical manifestations were similar to those of viral pneumonia (1). After sequencing and analyzing, researchers found a novel coronavirus and named it SARS-COV-2 (2). By April 28, 2021, the number of people infected with COVID-19 globally had exceeded 148 million, and the number of deaths had exceeded over 3.1 million (3). The outbreak has highlighted the inadequate global capacity to prevent, screen, and respond to unknown disease outbreaks, and the fight against infectious diseases remains one of the essential tasks of the 21st century.

A disease outbreak may start with a single infectious case that has not been presented for an extended period or caused by unknown agents (e.g., bacterium or virus) in the community or region, or the presence of a previously unknown disease (4). Most countries are now using syndromic surveillance as a method to identify potential public health threats. Syndromic surveillance collects data based primarily on non-specific health indicators and non-clinical indicators (5), and analyzes the time-space distortion of these data to achieve early detection and rapid response ability of public health events (6). However, the current syndromic surveillance systems belong to the passive surveillance mode and rely heavily on the reporters' identification of reportable diseases (7). Moreover, the surveillance objects are relatively single, unable to detect newly emerging outbreaks with previously unseen patterns of symptoms or other unexpected events of relevance to public health (8). Many medical staff members lack corresponding training and motivation toward emerging public health events in many regions, especially at the grassroots level (9). Therefore, relying only on syndromic surveillance is unable to detect unknown disease outbreaks, leading to the lag of response toward public health events (10).

Recently, researchers are focusing on using machine learning for pre-syndromic surveillance to identify relevant clusters of disease cases without pre-classification into syndromes. Lall et al. (11) and Walsh et al. (12) introduced a data-driven method to monitor the sudden increases in word frequency in the emergency department's chief complaint data to determine whether the text patterns in these data require further epidemiology investigation and continuous surveillance. Maurya et al. (13) proposed a Spatially Compact Semantic Scan (SCSS) method based on the Latent Dirichlet Allocation (LDA) model to identify spatially compact and temporally emerging topics in real-world text streams. Wu et al. (14) developed a deep learning framework to predict epidemiology profiles in the time series by using Recurrent Neural Network (RNNs) to obtain the long-term correlation in the data and Convolution Neural Networks (CNNs) to fuse information from data of different sources. Wang et al. (15) established a two-branch neural network structure to take both within-season and between-season observations as features. The framework enables detailed forecasting when high-resolution surveillance data is not available. Nobles et al. (16) developed a multidimensional semantic scanning method to study disease categories from data and monitor pre-syndromic diseases from the free-text emergency department chief complaints. However, machine learning has its unique challenges, such as data sparsity, lack of positive training samples (8), and ensemble prediction optimization (17). More importantly, a system needs to increase users' experience, make it closer to the medical staff members' preferences (18). The analysis and training process of machine learning is a “black box” to doctors, who can only get the accuracy of results, neglecting the importance of diagnostic thinking and medical knowledge transmission.

Therefore, instead of focusing on machine learning, this paper offered a new way to transform medical knowledge into structured data and form diagnostic rules, and use the method to explore unknown disease outbreaks detection.

## Materials and Methods

### Knowledge Collection and Organization

The modern medical model comprises three factors: biology, psychology, and society (19). Therefore, medical diagnostic knowledge collection should include clinical and non-clinical data, such as chief complaints, laboratory tests, auxiliary examinations, psychological states, working and living states, and epidemiological analysis of infectious diseases. This study collects diagnostic knowledge in the form of “concepts.” The concepts contain the names, the fields, and consist of simple concepts and compound concepts. The compound concepts are composed of simple concepts, while simple concepts refer to those basic concepts, which cannot be further subdivided. For example, “syndrome” is a compound concept formed by the combination of multiple “symptoms.” After establishing the diagnostic knowledge concepts, define the types and categories of concepts. According to the field attributes, classify concepts into numeric types, text types, sound and image types, and genetic types. Concepts belong to different categories: Combinations of fields define simple conceptual object categories; Combinations of simple conceptual categories define compound conceptual object categories.

### Define Concepts as the “Features”

Define the “Feature”: The Feature value represents the medical data's abnormal value with diagnostic significance for diseases. The diagnostic data contains chief complaints, laboratory findings, auxiliary examination results, biological detections, and psychological states. According to the medical definitions, the data has normal and abnormal values. By combining abnormal values with other factors, such as working and living states, doctors can conduct a comprehensive analysis and differential to give final diagnostic conclusions. The abnormal value can be either a value or a range of values. Through medical knowledge collections and organizations, classify and identify concepts belonging to the same category in disease diagnosis, according to the International Classification of Diseases Codes-10 (ICD-10) (20). The conditions that satisfy the classification are the Feature values of the concept. For example:

- The ICD-10 code: *R91* – “*Abnormal findings on diagnostic imaging of lung*,” according to the “abnormal findings,” can be divided into different characteristics, such as unilateral involvement and bilateral involvement. Thus, the classification of its Feature value consists of the classification conditions (abnormal findings) and mathematical operators: Abnormal findings = Unilateral involvement OR Abnormal findings = Bilateral involvement;
- The ICD-10 code: *R50.9* – “*Fever, unspecified*,” according to the “body temperature,” can be classified into low grade fever, moderate grade fever, high grade fever, and hyperpyrexia. The classification of its Feature value consists of the classification condition (body temperature, *T*) and mathematical operators: Low grade fever = 37.3°C ≤ *T* ≤ 38°C, Moderate grade fever = 38°C < *T* ≤ 39°C, High grade fever = 39°C < *T* ≤ 41°C, and Hyperpyrexia = 41°C < *T*;
- The ICD-10 code: *D70* – “*Leukopenia*” consists of combinations of multiple classification conditions (age, white blood cells (WBC) count) and mathematical operators: Adult leukopenia = Age ≥ 18 AND WBC count < 4 × 10^9^ per L;
- It can also consist of the specified machine learning classification algorithms through Big Data training and algorithmic classification results.

For presentation, define the Feature value as ***F***_(*x*)_, where *x* is the object, ***F*** means that *x* satisfies the classification condition specified by ***F***. ***F*** can be either a simple condition or a compound condition combined by connectives “AND” and “OR,” such as ***F*** = ***F***_1_ AND ***F***_2_,…, AND ***F***_*n*_, or ***F*** = ***F***_1_ OR (***F***_2_ AND ***F***_3_). When ***F*** = ***F***_1_ AND ***F***_2_,…, AND ***F***_*n*_, ***F***_(*x*)_ = ***F***_1(*x*)_ AND ***F***_2(*x*)_,…, AND ***F***_*n*(*x*)_.

On the concept category A, define a series of Feature values of A as ***F***_1_, ***F***_2_,…, ***F***_*n*_. Denote the set of all concepts on A as ***O***, and define the object set that satisfies the Feature *f* as ***O***_*f*_, as shown in [1]:

(1) $$\begin{array}{r} {\text{O}}_{f}=\left\{\;o\;|\;\forall o\in \text{O},{\;\text{F}}_{\left(o\right)}\;\right\} \end{array}$$

If $\text{O}={⋃}_{1}^{n}{\text{O}}_{F_{i}}$, then ***F***_1_, ***F***_2_,…, ***F***_*n*_ is a complete classification. Otherwise, it is an incomplete classification.

And for any *i, j* = 1,…, *n, i* ≠ *j*, if ***O***_*F*_*i*__ ∩ ***O***_*F*_*j*__ = ∅, then ***F***_1_, ***F***_2_,…, ***F***_*n*_ is a mutual exclusion Feature. Otherwise, it is a compatible Feature.

For concept category A, the Features set is the set of all Features ***F***_1_, ***F***_2_,…, ***F***_*n*_ defined on A. For presentation, define the Features set as FeatureSet (A), where A is the concept category, and FeatureSet (A) represents the set of all Feature values defined on the concept category A.

For the compatible Features, specify the matching methods as priority match and full match:

- Priority match refers to matching according to the sequence and return as a unique Feature;
- Full match refers to returning with all Features that successfully matched.

### Define Inference Rules Based on the Features

Define the Features as the premises (P) of inference rules, the conclusions (or actions) of inference rules is Q. Hence, the new inference rule can be expressed as [2]:

(2) $$\begin{array}{r} P:{\text{F}}_{1\left(\chi_{1}\right)}\;⋀{\;\text{F}}_{2\left(\chi_{2}\right)}\;⋀\ldots ⋀\;{\text{F}}_{n\left(\chi_{n}\right)}\to Q \end{array}$$

Where **F**_1(χ_1_)_, **F**_2(χ_2_)_, …, **F**_*n*(χ_*n*_)_ are the Features. The Features have truth and false values. The truth-value represents that the Feature meets conditions. If **F**_1(χ_1_)_, **F**_2(χ_2_)_, …, **F**_*n*(χ_*n*_)_ are all-true, then obtain the corresponding conclusion Q. When defined the Feature ***F***_(*x*)_, it is known that ***F***_(*x*)_ can be either a simple Feature or a compound Feature, which consists of multiple simple Features combined by “AND” or “OR.” Therefore, any Feature **F**_*n*(χ_*n*_)_ in inference rules can also be a compound Feature.

### Build Knowledge Bases to Store Inference Rules

**Figure 1:**
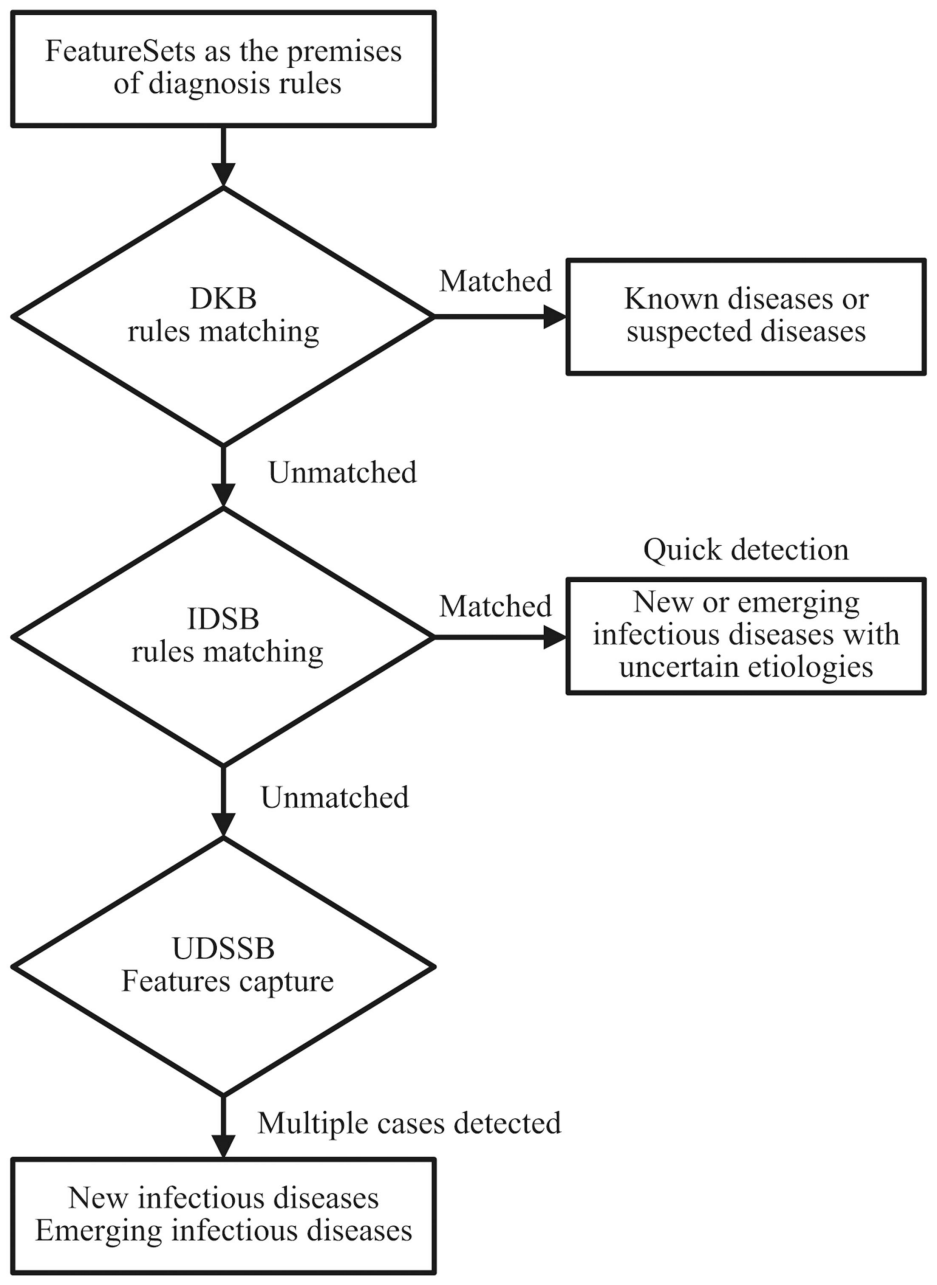
Diagnosis and detection process schematic diagram of the target cases; DKB, Diagnosis Knowledge Base; IDSB, Infectious Diseases Surveillance Base; UDSSB, Unknown Diseases Syndromic Surveillance Base.

As shown in Figure 1, define the diagnostic data of the target case as the FeatureSets, such as chief complaints, radiography findings, laboratory findings, and treatment outcomes. Use the FeatureSets as the antecedents (or premises) of inference rules and carry out the following processes:

1. Build a Diagnosis Knowledge Base (DKB) with the diagnostic knowledge collected and organized by professional medical teams, to form inference rules composed of the FeatureSets and consequents (diseases). Use the target case's Feature values as the antecedent and match with the rules stored in the DKB: If the match succeeds, it indicates that the target case is diagnosed as a known disease. If certain antecedent nodes are missing, for example, an absence of laboratory findings, the consequent of its relevant rule can be defined as a suspected disease. When the antecedent failed to match in the DKB, perform step 2;
2. Build an Infectious Diseases Surveillance Base (IDSB) to store surveillance guidelines, which consist of quick detection rules of new or emerging infectious diseases (EID) with different syndrome groups. Match the antecedent with the rules stored in the IDSB: If the match succeeds, it indicates that the target case may a new or emerging infectious disease, the etiology remains uncertain. The system will send a notification to the local centers for disease control and prevention (CDC) automatically, and staff members from the local CDC can carry out epidemiological surveys in response. If the system detects multiple cases within a short period, it indicates that these cases are possibly the aggregation of a certain infectious disease. When the antecedent again failed to match in the IDSB, perform step 3;
3. Build an Unknown Diseases Syndromic Surveillance Base (UDSSB) to capture the diagnostic FeatureSets of the target case and conduct statistical analysis: This situation indicates that the target case may be an unknown disease (non-infectious) or an EID, or the knowledge of the DKB and IDSB is insufficient and needs a supplement. In the UDSSB, if the system detects a sudden cluster of certain FeatureSets within a short period, it indicates that these cases are possibly the aggregation of infectious disease, the system will send different levels of alerts according to the statistical results. If the system does not detect abnormal clusters, store the captured Features in the UDSSB with continuous surveillance. Simultaneously, the researchers can conduct an early epidemiological survey of the target case to collect data.

## Results

Over the past decade, many countries and regions have established surveillance guidelines for EID, e.g., the Global Disease Detection International Emerging Infections Program (21). These surveillance guidelines cover different syndrome groups, such as respiratory, gastrointestinal, acute jaundice, and infectious neurological diseases. With the help of these guidelines, countries can perform quick responses to infectious disease threats (22).

This study used the surveillance guideline for pneumonia with uncertain etiologies in China to evaluate the feasibility of the proposed method. In 2007, the Chinese Ministry of Health established the “National Program for Surveillance, Screening, and Management, for the cases of Pneumonia with Uncertain Etiologies (PUE) (23),” which stipulates that:

1. The case of PUE refers to the following four criteria, and cannot be diagnosed as other known diseases:
  - Fever (axillary temperature ≥ 38°C);
  - Diagnostic imaging of lung has pneumonia or ARDS characteristics;
  - In the early stage of the disease, the total number of WBC decreased or remained normal, or the classified count of lymphocytes decreased;
  - After standard treatment with antibiotics for 3–5 days, there was little improvement or revealed progressive disease exacerbation.
2. Aggregation of PUE cases: two or more cases with epidemiological correlations occurred within 2 weeks.

This study suggested that the guideline to some extent can be used to detect certain infectious disease outbreaks, mainly with respiratory syndromes, such as Severe Acute Respiratory Syndrome (SARS), Middle East Respiratory Syndrome (MERS), and COVID-19.

### Define the Features and Surveillance Rules

For adult patients, to meet the criteria of the guideline, the clinical characteristics should include:

1. Fever (the most common symptom, oral temperature ≥ 37.3°C);
2. Abnormalities on diagnostic imaging of lung with unilateral or bilateral involvement;
3. The WBC count remains normal or decreased, or the lymphocyte count decreased;
4. Resistance to antibiotic treatments;
5. Cannot be diagnosed as other known diseases.

By defining criteria 1-4 according to the ICD-10 codes (20), obtain the Features (sets) of PUE cases, as shown in Table 1. Define the surveillance rule of the PUE case based on the defined Features (sets) and store it in the IDSB, as shown in rule [3]:

(3) $$\begin{array}{r} {\text{F}}_{\left(R50.9\right)}⋀\;{\text{F}}_{\left(R91\right)}⋀\;\text{F}={\text{F}}_{\left(D70\right)}OR{\text{F}}_{\left(D72.8\right)}⋀\;{\text{F}}_{\left(U83.9\right)}\to \\ \text{PUE case} \end{array}$$

Where ***F***_(*R*50.9)_ ⋀ ***F***_(*R*91)_ ⋀ ***F*** = ***F***_(*D*70)_ OR ***F***_(*D*72.8)_ ⋀ ***F***_(*U*83.9)_ is the antecedent (or premise) of the surveillance rule, “PUE case” is the consequent (or conclusion).

**Table 1:** Feature definitions of pneumonia with uncertain etiologies cases according to the concept categories and the International Classification of Diseases codes-10.

**Categories**	**ICD-10 codes and definitions**	**Features (sets)**
Symptoms	R50.9: Fever, unspecified	Low grade fever = 37.3°C ≤ T ≤ 38°C Moderate grade fever = 38°C < T ≤ 39°C High grade fever = 39°C < T ≤ 41°C Hyperpyrexia = 41°C < T
Chest radiography findings	R91: Abnormal findings on diagnostic imaging of lung	Abnormal findings = Unilateral involvement OR Bilateral involvement
Laboratory findings	D70: Leukopenia	Adult leukopenia^*^= Age ≥ 18 AND WBC count <4.0 × 10^9^ per L
	D72.8: Lymphopenia	Adult lymphopenia^*^= Age ≥ 18 AND lymphocyte count <1.0 × 10^9^ per L
Treatment outcomes	U83.9: Resistance to unspecified antibiotic	Antibiotic resistance = No significant effects OR Progressively exacerbated

*ICD-10 = International Classification of Diseases codes-10; T = Oral temperature; WBC = White Blood Cells*.

* *Varies based on the different definition criteria*.

### SARS Outbreak Detection

Liu et al. (24) reviewed medical records for a total number of 36 patients with probable SARS from April 27 to May 24, 2003, in Taiwan, as shown in Table 2. According to the research, all patients presented fever (36/36, 100%) on admission, the mean temperature was 38.8°C (38.0–40.1°C). Other common symptoms include chills (75%), non-productive cough (44.4%), diarrhea (41.7%), myalgia (38.7%), and productive cough (19.4%). During hospitalization, the chest radiographs showed unifocal or multifocal infiltration in 35 (97.2%) patients, the laboratory findings showed leukopenia in 17 (47.2%) patients and lymphopenia in 33 (91.7%) patients. All patients received antibiotic therapy (macrolide and fluoroquinolone) and no specific antibiotic appeared to be independently effective.

**Table 2:** Characteristics summary of probable Severe Acute Respiratory Syndrome (SARS) patients according to the surveillance guideline for pneumonia with uncertain etiologies, from April 27 to May 24, 2003, in Taiwan (24).

**Characteristics**	Proportion, n/N^*^ (%)	
Symptoms:	–	
Fever	36/36 (100%)	
Chest radiography findings:	–	
Unifocal infiltration	13/36 (36.1%)	35/36 (97.2%)
Multifocal infiltration	22/36 (%)	
Laboratory findings:	–	
Leukopenia: WBC count <3.5 × 10^9^ per L	17/36 (47.2%)	
Lymphopenia: Lymphocyte count <1.0 × 10^9^ per L	33/36 (91.7%)	
Treatments:	–	
Macrolide and Fluoroquinolone	36/36 (100%)	

*WBC = White Blood Cells*.

* *N is the total number of patients with available data*.

The detection process starts with data input. Doctors input the patient's chief complaints, laboratory findings, radiography findings, treatments, and outcomes data according to the system's prompts, forms the FeatureSets of the target case. For SARS cases, the system selected the FeatureSet ***F***_(*R*50.9)_ of the most common symptom fever as the root node, radiographic abnormalities ***F***_(*R*91)_, hematological abnormalities lymphopenia ***F***_(*D*72.8)_, and antibiotic therapy outcomes ***F***_(*U*83.9)_ as the children nodes 2-4 to set up the premise [P (SARS)] of the diagnosis rule, as shown in premise [4]:

(4) $$\begin{array}{r} P\left(SARS\right)={\text{F}}_{\left(R50.9\right)}⋀\;{\text{F}}_{\left(R91\right)}⋀\;{\text{F}}_{\left(D72.8\right)}⋀\;{\text{F}}_{\left(U83.9\right)} \end{array}$$

The system carried out rule matching in the DKB based on premise [4], and could not match it with any stored rules, thus concluded “Unmatched in the DKB” after searching, as shown in [5]:

(5) $$\begin{array}{r} P\left(SARS\right):{\text{F}}_{\left(R50.9\right)}⋀\;{\text{F}}_{\left(R91\right)}⋀\;{\text{F}}_{\left(D72.8\right)}⋀\;{\text{F}}_{\left(U83.9\right)}\to \\ \text{Unmatched in the DKB} \end{array}$$

Where ***F***_(*R*50.9)_ ⋀ ***F***_(*R*91)_ ⋀ ***F***_(*D*72.8)_ ⋀ ***F***_(*U*83.9)_ is the premise, the matching result is “Unmatched.” It indicated that the diagnostic knowledge in the DKB could not diagnose the target cases as any known diseases. Therefore, input the target cases into the IDSB to carry out rule matching. As shown in rule [6], the system searched in the IDSB and detected that premise [4] matched with the premise of rule [3], thus concluded: The disease of the patients was a kind of PUE, the cause of pneumonia remained uncertain.

(6) $$\begin{array}{r} P\left(SARS\right):{\text{F}}_{\left(R50.9\right)}⋀\;{\text{F}}_{\left(R91\right)}⋀\;{\text{F}}_{\left(D72.8\right)}⋀\;{\text{F}}_{\left(U83.9\right)}\to \\ \text{PUE case} \end{array}$$

Where ***F***_(*R*50.9)_ ⋀ ***F***_(*R*91)_ ⋀ ***F***_(*D*72.8)_ ⋀ ***F***_(*U*83.9)_ is the premise, “PUE case” is the conclusion. The system reported to the local CDC automatically. The matching accuracy of the IDSB was calculated by the proportions of each inference node and the result was 89.1% (100 × 97.2 × 91.7 × 100%). When the system detected multiple PUE cases within 2 weeks, the local CDC would notice that the target cases were possibly an aggregation of PUE cases. For the rest 10.9% of target cases that failed to match in the IDSB, the system captured the FeatureSets of the target cases, including other common symptoms into the UDSSB, and carried out similar case searches. If the system found similar cases that occurred in a region within 2 weeks, the system would send a notification to the local CDC, indicating that these cases were possibly the aggregation of an unknown or emerging infectious disease, thus the local CDC would respond by carrying out an early epidemiological investigation. If the system did not find similar cases, store the captured FeatureSets in the UDSSB with continuous surveillance.

### MERS Outbreak Detection

This study reviewed the clinical and outcomes characteristics of confirmed cases of MERS from the prior studies (25–27). After statistically integrated their findings, this study summarized the clinical and outcomes data of MERS patients according to the surveillance guideline for the PUE, as shown in Table 3. The number of patients was 105 in total and the admission data was from September 1, 2012, to December 2018. The most common symptoms on admission included fever (85.7–98%, the median temperature was 37.5°C), fever with chills or rigors (87%), cough (83–100%), shortness of breath (57.1–72.5%), myalgia (14.3–32%), diarrhea (13.7–26%), and sore throat (13.7–21%). The chest radiographs showed abnormalities with pneumonia evidence among 90–100% of patients. The laboratory findings showed 14–42.9% of patients presented leukopenia, and 34–100% of patients presented lymphopenia. The patients received various antibiotics for treatments, including Azithromycin, Piperacillin/Tazobactam (Tazocin), Ceftriaxone, and Vancomycin, the effects were not obvious.

**Table 3:** Characteristics summary of Middle East Respiratory Syndrome (MERS) patients according to the surveillance guideline for pneumonia with uncertain etiologies, from September 1, 2012, to December 2018, in Saudi Arabia (25–27).

**Characteristics**	Proportion, n/N^*^ (%)
Symptoms	–
Fever	85.7–98%
Chest radiography findings	–
Abnormalities with pneumonia evidence	90.2–100%
Laboratory findings	–
Leukopenia	5–42.9%
Lymphopenia	34–100%
Treatments	–
Antibiotic therapy	100%

* *N is the total number of patients with available data*.

For MERS detection, the system set up the premise [P (MERS)] of the diagnosis rule. After matching in the DKB and IDSB, the system could not diagnose the target cases as any specific known diseases, but the P (MERS) met the criteria for PUE cases, as shown in rule [7]. Therefore, the system reported to the local CDC automatically.

(7) $$\begin{array}{r} P\left(MERS\right):{\text{F}}_{\left(R50.9\right)}⋀\;{\text{F}}_{\left(R91\right)}⋀\;{\text{F}}_{\left(D72.8\right)}⋀\;{\text{F}}_{\left(U83.9\right)}\to \\ \text{PUE case} \end{array}$$

The matching accuracy of the IDSB varied between 26.3% and 98%. When the system detected multiple cases within 2 weeks, the local CDC would notice that the target cases were possibly the aggregation of PUE cases. Simultaneously, for the rest 2–73.7% of target cases that failed to match in the IDSB, the system captured the FeatureSets of the target cases, including other common symptoms into the UDSSB, and carried out similar case searches. If the system found similar cases that occurred in a region within 2 weeks, the local CDC would carry out an early epidemiological investigation. If the system did not find similar cases, store the captured FeatureSets in the UDSSB with continuous surveillance.

### Early COVID-19 Outbreak Detection

Huang et al. (1) reviewed clinical charts, nursing records, laboratory findings, and chest x-rays for early patients with laboratory-confirmed COVID-19 infection in Wuhan, China. The number of the patients was 41 and the admission data was from December 16, 2019, to January 2, 2020. As shown in Table 4, 40/41 patients (98%) presented fever (highest temperature ≥ 37.3°C) on admission, abnormalities on chest radiography were detected among all patients (bilateral involvement, 98%). The laboratory findings showed 10/40 (25%) patients presented leukopenia (WBC count < 4.0 × 10^9^ per L) and 26/41 (63%) patients presented lymphopenia (lymphocyte count < 1.0 × 10^9^ per L). The total number of patients presented leukopenia or lymphopenia was 35/41 (85%). All patients (41/41, 100%) were given antibiotic treatments and no specific therapy showed independent effectiveness.

**Table 4:** Characteristics summary of early COVID-19 patients according to the surveillance guideline for pneumonia with uncertain etiologies, from December 16, 2019, to January 2, 2020, in Wuhan (1).

**Characteristics**	**Statistical results**	
Symptoms	n/N^*^ (%)	
Fever	40/41 (98%)	
Highest temperature,°C	–	
37.3–38.0	8/41 (20%)	
38.1–39.0	18/41 (44%)	
>39.0	14/41 (34%)	
Chest radiography findings	n/N (%)	
Bilateral involvement	40/41 (98%)	
Laboratory findings	n/N (%)	
Leukopenia: WBC count <4.0 × 10^9^ per L	10/40 (25%)	35/41 (85%)
Lymphopenia: Lymphocyte count <1.0 × 10^9^ per L	26/41 (63%)	
Treatments	n/N (%)	
Antibiotic therapy	41/41 (100%)	
Time course	Days, Median	
Time from onset of symptoms	–	
To first hospital admission	7.0 (4.0–8.0)	
To ARDS	9.0 (8.0–14.0),	
To mechanical ventilation	10.5 (7.0–14.0)	
To ICU admission	10.5 (8.0–17.0)	

*WBC = White Blood Cells; ARDS = Acute Respiratory Distress Syndrome; ICU = Intensive Care Unit*.

* *N is the total number of patients with available data*.

**Figure 2:**
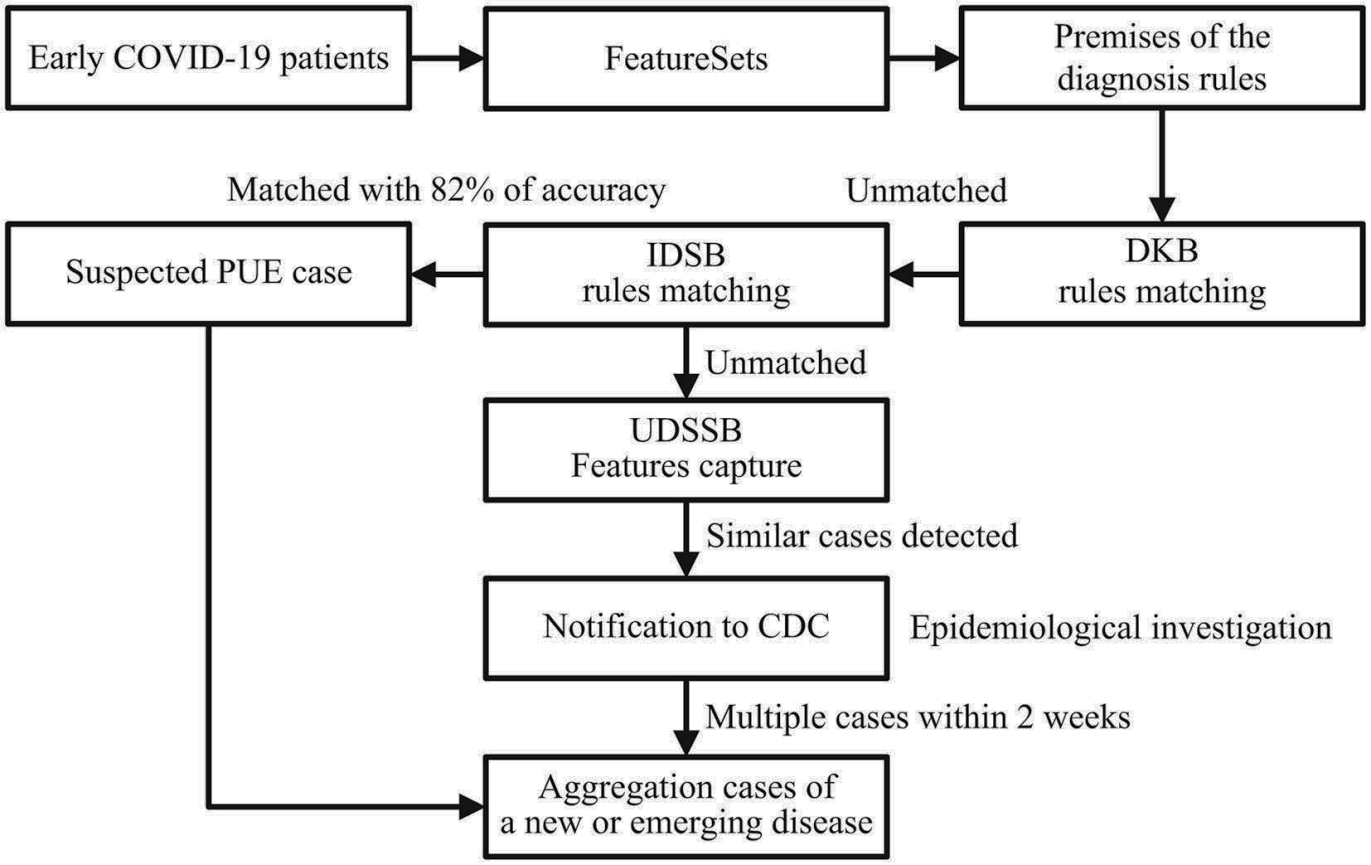
Detection process schematic diagram of the early COVID-19 cases in Wuhan, China; DKB, Diagnosis Knowledge Base; IDSB, Infectious Diseases Surveillance Base; PUE, Pneumonia with Uncertain Etiologies; UDSSB, Unknown Diseases Syndromic Surveillance Base; CDC, Centers for Disease Control and Prevention.

As shown in Figure 2, doctors first input the chief complaints, radiography findings, laboratory findings, and treatment outcomes of the patients to the system, formed the FeatureSets of the target cases. The system selected ***F***_(*R*50.9)_ as the root node, ***F***_(*R*91)_, ***F*** = ***F***_(*D*70)_ OR ***F***_(*D*72.8)_, and ***F***_(*U*83.9)_ as the children nodes to set up the premise [P (COVID-19)] of the diagnosis rule, as shown in premise [8]:

(8) $$\begin{array}{r} P\left(COVID-19\right):{\text{F}}_{\left(R50.9\right)}⋀\;{\text{F}}_{\left(R91\right)}⋀\;\text{F}={\text{F}}_{\left(D70\right)}OR{\text{F}}_{\left(D72.8\right)}\\ ⋀\;{\text{F}}_{\left(U83.9\right)} \end{array}$$

Through matching P (COVID-19) [8] with the rules in the DKB and IDSB, the system could not diagnose the patients as any known diseases, whereas P (COVID-19) [8] matched with the premise of rule [3], as shown in rule [9]. Therefore, the patients met the criteria for the PUE cases, the system reported to the local CDC automatically.

(9) $$\begin{array}{r} P\left(COVID-19\right):{\text{F}}_{\left(R50.9\right)}⋀\;{\text{F}}_{\left(R91\right)}⋀\;\text{F}={\text{F}}_{\left(D70\right)}\;OR\;{\text{F}}_{\left(D72.8\right)}\\ ⋀\;{\text{F}}_{\left(U83.9\right)}\to \text{PUE case} \end{array}$$

The matching accuracy of the IDSB was 82% (98 × 98 × 85 × 100%). Additionally, 27/41 (66%) patients had direct exposure to the Wuhan Huanan seafood market, and their date of illness onset was from December 10 to 31, 2019 (1). When the system detected multiple cases within 2 weeks, the system would send several notifications to the local CDC, thus the staff members would consider these cases as the aggregation of PUE and perform an early epidemiological survey in response. The early COVID-19 patients' data showed that except for the fever, the patients had other common symptoms at the onset of illness, such as cough (76%), dyspnea (55%), myalgia or fatigue (44%), and sputum production (28%) (1). For the rest 18% of patients that failed to match in the IDSB, the system would capture the FeatureSets of their clinical characteristics into the UDSSB and conduct similar case searches. When the system detected multiple cases that occurred in Wuhan within 2 weeks, the local CDC would receive notifications and carry out an early epidemiological survey in response.

## Discussion and Conclusions

According to the early COVID-19 data in Wuhan, the median time from onset of symptoms to first hospital admission of 41 patients was 7.0 days (4.0–8.0), to acute respiratory distress syndrome (ARDS) was 9.0 days (8.0–14.0), to mechanical ventilation was 10.5 days (7.0–14.0), and to intensive care unit (ICU) admission was 10.5 days (8.0–17.0) (1). The data of 41 patients represented the real situation and response speed of local citizens when an unknown disease outbreak occurred in Wuhan, December 2019, and from hospital admission to local CDC responses, the whole process could be easily disturbed by human factors. Although the method proposed by this study cannot reduce the timeliness of patients from onset of symptoms to hospital admission, it can assist doctors in differential diagnosis after inputting the clinical characteristics data of patients, hence shortening the ambiguous process of diseases from unknown to known and providing early clues to CDC for carrying out epidemiological investigations. Once doctors input the patients' diagnostic data, the system will start to match the target case with the knowledge bases, some of the data may need a day or two to collect, such as radiography findings and treatment outcomes. After detecting two or more cases in the IDSB and UDSSB, the system will report to CDC automatically. Therefore, based on the early COVID-19 data in Wuhan, 2019, this study estimates that with the system's help, the detection and notification timeliness of the early COVID-19 pandemic can be reduced to 7.0–10.0 days, and the detection process can avoid being disturbed by human factors.

Since the outbreak of the COVID-19 pandemic in 2019, various studies have focused on collecting and delineating the demographic, clinical, and outcomes data on confirmed cases, some of the patients' numbers are over thousands (28, 29). However, the data from subsequent studies were obtained based on the increasing number of new scientific findings, as well as increasing awareness of the pandemic by hospitals, CDCs, and governments, which resulted in the strengthened prevention and control measurements of the pandemic. For example, for the confirmed cases recruited after January 1, 2020, in China, 31.3% of cases had recently been to Wuhan, 71.8% of cases had contact with people from Wuhan (28), hence other detection methods, such as cluster screening, molecular tests, diagnostic imaging, and biochemical detections can be more effective after the initial outbreak (30, 31). Moreover, the appearance of asymptomatic infections since 2020 has reduced the strength of using clinical characteristics for detection (29, 32, 33). Therefore, this study suggests that the data of the initial 41 patients with laboratory-confirmed COVID-19 infection in Wuhan is more suitable for evaluating our method.

The matching accuracy of the IDSB represents the system's ability to quickly detect suspected PUE cases according to the surveillance guideline stored in the IDSB, and it was calculated based on the proportions of each data presented in the prior studies. The surveillance rule of the PUE cases has four inference nodes, the proportion on each node represents the accuracy of the system to successfully match the node with patients. Multiply the proportions of each inference node, the final results represent the accuracy of the system to match the target cases with the surveillance rule of PUE cases in the IDSB. The empirical study of the MERS pandemic showed a higher matching accuracy fluctuation (26.3–98%) of the IDSB, this is due to the surveillance guideline we used has limited the detection scale of MERS cases, as well as the high fluctuation of patients' lymphopenia data (34-100%) (25–27). However, the UDSSB was designed to capture the FeatureSets of the remaining patients and ensure the overall detection accuracy of the system. After detecting multiple similar cases within a short period in the UDSSB, the system would send notifications to CDC, indicating the suspected aggregation of an unknown or emerging infectious disease. On the other hand, this study suggests that the matching accuracy is not the key factor for evaluating the feasibility of the method. The matching accuracy is not equal to the detection accuracy, which can be affected by several problems, such as the system's ability to detect an unknown disease, which may present similar presentations of a known disease, and the ability to diagnose a known disease, which may present nonstandard presentations. In this study, the system used medical knowledge in the DKB to solve such problems. To increase the detection accuracy of the system, we need to increase the width and depth of the system's DKB. The width of the knowledge base represents the system's ability to diagnose different types of diseases, the depth represents the system's ability to diagnose complex cases. Therefore, to improve the feasibility of the method, medical knowledge should be constantly added to the knowledge base through daily diagnostic activities.

Our study has the following novelties. First, this is the first attempt to use a new Feature-based knowledge representation and reasoning approach to organize medical knowledge and detect unknown disease outbreaks. Unlike the other artificial intelligence methods that facing unique challenges such as data sparsity and lack of training data sets, our method will enable medical experts to continually input their professional knowledge into the system and form diagnostic rules with different criteria, hence adapt the system in response to the potential public threats under the global scenario. More importantly, for most doctors, especially those from remote and underdeveloped areas, this method can extend their expertise and experience, improve their abilities in clinical thinking and differential diagnosis. Second, by matching with diagnosis rules in the DKB, the system can assist doctors in their daily diagnosis and distinguish emerging diseases from common diseases, thus the surveillance mode can change from passive to active. Additionally, the method combined two knowledge bases with an additional syndromic surveillance base to ensure the overall detection accuracy and reduce the misdiagnosis rate.

The most noticeable limitation of this study is the lack of clinical characteristics and treatment outcomes data on the initial patients, who were the earliest patients confirmed infected by SARS and MERS coronavirus. An empirical study with initial infected patients' data can better evaluate the feasibility of our method for unknown disease outbreak detection. Secondly, as a pilot study, it has not yet compared the empirical results with other Artificial Intelligence approaches. We put more emphasis on the description of the Features' construction, and limitations may occur in data integrity, consistency, and validity. Thirdly, to improve the feasibility of the method, medical knowledge shall be constantly added to the DKB through daily diagnostic activities. Besides, the surveillance guideline our study used was established by the Chinese government in 2007, the definitions of the PUE case had limited the selection of outbreaks based on different epidemiological, demographic, and clinical characteristics in the empirical study, and need further supplements.

Future research work includes increasing the number of patients, adding comparisons between different syndrome groups, regions, and populations. Additionally, we are currently developing a new reasoning method combined with the Feature-based knowledge representation. The reasoning method will focus on several issues, including distinguishing different diseases from similar combinations of phenomena, getting multiple possible results from a group of phenomena, sorting the results according to certain rules, and screening the results based on the information input by doctors. The reasoning method will be designed with an empirical study to test the feasibility of unknown or complex disease detection in different circumstances.

In conclusion, this study proposed a new Feature-based knowledge representation and reasoning model under the main background of the COVID-19 pandemic in 2019 and used the model to explore the detection of unknown disease outbreaks. By defining the abnormal diagnostic data as the Features, the medical knowledge can transform into structured data and use the Features as antecedents of the inference rules, which can then match with the system's knowledge bases to detect unknown or emerging disease outbreaks. The results of the empirical study demonstrate that by combining with suitable surveillance guidelines, the method proposed in this study is capable to detect outbreaks of SARS, MERS, and COVID-19 pandemics. Furthermore, the method will enable medical experts to input their professional knowledge into the system's knowledge bases, and by continuously accumulating the diagnostic knowledge and surveillance guidelines, the system's detection accuracy and scale will be improved. More importantly, the knowledge representation approach can activate the sharing and transmission process of medical knowledge, thus accelerate the accumulation speed of doctors' knowledge and experience, minimize the time of training an outstanding medical expert.

## Data Availability Statement

The original contributions presented in the study are included in the article/supplementary material, further inquiries can be directed to the corresponding author/s.

## Ethics Statement

Ethical review and approval was not required for the study on human participants in accordance with the local legislation and institutional requirements. Written informed consent for participation was not required for this study in accordance with the national legislation and the institutional requirements.

## Author Contributions

RF, QH, and YJ shared research design and overall supervision of the process. RF took responsibility for putting together the final manuscript, all authors gave feedback on this process. RF and QH were responsible for the methods design and empirical study of the study. YJ provided additional support with data analysis. All authors contributed to the article and approved the submitted version.

## Conflict of Interest

The authors declare that the research was conducted in the absence of any commercial or financial relationships that could be construed as a potential conflict of interest.
